# Adaptive lifestyle of bacteria determines phage-bacteria interaction

**DOI:** 10.3389/fmicb.2022.1056388

**Published:** 2022-12-06

**Authors:** Laura Ulrich, Christoph Giez, Leon X. Steiner, Ute Hentschel, Tim Lachnit

**Affiliations:** ^1^Zoological Institute, Christian-Albrechts-Universität, Kiel, Germany; ^2^RD3 Marine Ecology, RU Marine Symbioses, GEOMAR Helmholtz Centre for Ocean Research, Kiel, Germany

**Keywords:** bacteriophage, phage infection, *Curvibacter*, biphasic life cycle, microbiota, bacterial adaptation

## Abstract

Bacteriophages and their interactions with microbes are not well understood. As a first step toward achieving a better understanding, we isolated and sequenced the *Curvibacter* phage PCA1 for the purpose of eliminating *Curvibacter* sp. AEP1.3, the main colonizer of *Hydra vulgaris* AEP. Our experiments showed that PCA1 phage caused a strong, virulent infection only in sessile *Curvibacter* sp. AEP1.3 but was unable to infect planktonic and host-associated bacterial cells of the same strain. In an effort to investigate this phenomenon, we compared sessile, planktonic, and host-associated bacteria *via* RNA sequencing and found that all three states differed significantly in their expression patterns. This finding led us to propose that the adaptive lifestyle of *Curvibacter* sp. AEP1.3 results in varying degrees of susceptibility to bacteriophage infection. This concept could be relevant for phage research and phage therapy in particular. Finally, we were able to induce phage infection in planktonic cells and pinpoint the infection process to a membrane protein. We further identified potential phage-binding protein candidates based on expression pattern analysis.

## Introduction

Bacteriophages are highly diverse and abundant, being one of the most dominant entities on this planet with up to 10^54^ particles in the biosphere (Hatfull and Hendrix, 2011). Phages can infect bacteria by injecting their genetic material into a host, after which the genetic material is either integrated into the host genome or translated to produce phage particles. The latter is particularly characteristic of lytic phages. Lytic phages are typically used in therapeutic applications due to their ability to lyse bacteria and thus eliminate pathogenic ones (Weinbauer, 2004). This approach could not only be utilized to remove pathogenic bacteria but also to selectively modify bacterial community composition.

Bacterial communities are complex and colonize diverse habitats. In recent years, it has become apparent that they associate with multicellular organisms and are part of a multifaceted microbiota alongside viruses, archaea, fungi, and other protists (Davenport et al., 2017; Leftwich et al., 2020; Matijašić et al., 2020). While bacteriophages can only interact with prokaryotes, they are still capable of strongly impacting community compositions and thus affect homeostasis (Lourenço et al., 2022). The importance of a functional microbiota is already apparent during the early stages of human development (Zhuang et al., 2019) and is furthermore instrumental in immune cell maturation, antiviral defense (Gonzalez-Perez et al., 2016), digestion (Bäckhed et al., 2005), maintenance of physiological homeostasis (Sawa et al., 2011; Hergott et al., 2016), and protection against pathogens (Lunjani et al., 2019).

A mutually beneficial interaction between host and microbiota is naturally not limited to humans, as there are multiple examples of such mutualistic interactions, e.g., corals that are shielded from environmental stress, toxins, and pathogens through their microbiota (Peixoto et al., 2021) and aphids whose endosymbiotic bacteria protect them from parasites and heat stress (Zytynska, 2019).

Established methods to investigate the microbiota include but are not limited to usage of antibiotics, germ-free animals, and sequencing (Kennedy et al., 2018). These methods either rely on removing all associated bacteria or observing microbial communities as a whole, which limits functional analysis to inferences based on the entire microbial community. We want to propose bacteriophages as an alternative solution, since bacteriophages are able to specifically target and eliminate a bacterial strain. Therefore, phages could make it possible to conduct functional research on the impact a single bacterial strain has on an organism and its microbiota, by removing that single strain without disturbing the remaining microbiota (Paule et al., 2018). An additional advantage of phages is that they have rarely been observed to cause adverse effects, since they are able to bind specifically to bacterial receptors (Harada et al., 2018; Putra and Lyrawati, 2020), while antibiotics reportedly bind to 80S ribosomes, altering translation (Prokhorova et al., 2017) and inhibiting eukaryotic cell growth (Neftel and Hübscher, 1987). Lastly, phages are highly diverse and abundant with up to 10^31^ phage particles in the biosphere (Hatfull and Hendrix, 2011; Dion et al., 2020) and can further be biogenetically engineered to optimize infectivity and host range by homologous recombination (Gordillo Altamirano and Barr, 2019), random guanine alkylation (Favor et al., 2020), or by using CRISPR-Cas systems (Pires et al., 2016).

Since application of phages to microbiota research is not well established, we chose the freshwater polyp *Hydra vulgaris* AEP (Augustin et al., 2012; Galliot, 2012) as our model. A mucus layer outside the cnidarian’s ectodermal epithelium (Bosch et al., 2010) serves as the sole point of interaction between *Hydra* and its microbiota (Pietschke et al., 2017), further facilitating the study of microbiota-host interactions.

In the present study, we focus on *Curvibacter* sp. AEP1.3, *Hydra’s* main colonizer, which represents approximately 75% of its whole microbiota (Fraune et al., 2015). This Betaproteobacterium protects its host against fungal infection together with *Duganella sp.* C1.2 (Li et al., 2015). Due to the high abundance of *Curvibacter sp.* AEP1.3 and its known protective function, it stood out as a good candidate to be targeted by phage intervention. Thus, we isolated the bacteriophage PCA1, characterized it, and tested its ability to infect and eliminate *Curvibacter sp.* AEP1.3 *in vitro* and on *Hydra.*

## Materials and methods

### Sample collection

In May 2020, we sampled 1 l of lake water from the “Bioturm” pond near the Zoological Institute in Kiel and warmed it up to room temperature (RT) overnight. Water samples were divided into two 500 ml flasks, one was enriched with R2A broth (Neogen®). We incubated both flasks at RT and 150 rpm overnight, before filtering the liquid (grade 595 1/2, Whatman®), and transferring it into 250 ml centrifuge bottles (Beckman Coulter, Polycarbonate). Samples were centrifuged at 10,000 rpm for 30 min using an Avanti JXN centrifuge (Beckman Coulter, JA-14 fixed angle rotor) and supernatant filtered using 0.2 μm filters (Whatman®, Sigma-Aldrich) to remove bacteria. We dissolved 10% (w/v) Polyethylene glycol (Sigma-Aldrich) in the supernatant, while it rested on ice for 2 h, after which we centrifuged the samples at 10,000 rpm for 30 min. Pellets were re-suspended using 3 ml SM-buffer (50 mM Tris–HCL, 100 mM NaCl, 8 mM MgSO4 at pH 7.5) in 15 ml Falcon tubes (Sigma-Aldrich). Finally, 10% (v/v) of chloroform were added to the phage solution and stored it at 4°C.

### Phage isolation

5 × 20 μl of phage solution (see above) were spotted on top of 4 ml overlay agar (1.2 g Neogen® R2A broth and 1.6 g agarose in 400 ml autoclaved, ultrapure water and stored at 60°C), containing 1 ml of *Curvibacter* sp. AEP1.3 culture at 0.2 OD. All cultures were prepared under sterile conditions using a lamellar flow bench (Biological safety cabinet, Thermo Scientific). Overlay agar plates were incubated at 18°C. Once phage plaques became visible, we cut them out and placed them into 2 ml liquid *Curvibacter* sp. AEP1.3 culture. We incubated our samples overnight at 18°C and filtered them using 0.2 μm pore filters to remove bacteria. Resulting phage mixture was diluted in R2A medium and 10 μl of each dilution placed into a mixture containing 4 ml overlay agar and 1 ml liquid *Curvibacter* sp. AEP1.3 (OD 0.2). The overlay mixture was plated out and the resulting plates incubated at RT until plaque-forming units (PFU) became visible. We cut out single PFU from each plate and amplified the phage isolates in 1 ml liquid *Curvibacter sp.* AEP1.3 culture overnight. After sterile filtration and PFU counts, we realized that amplification efficiency was low in liquid culture and thus mixed 20 μl of phage solution into 180 μl of liquid *Curvibacter sp.* AEP1.3 culture, which we plated onto R2A agar plates. Once plaques became visible, we rinsed off both phages and bacteria with 5 ml R2A medium per plate. Bacterial cells were removed by centrifugation and filtration (see above). Half of the amplified phage solution was used for DNA Extraction (see below), while the other half was conserved in 10% (v/v) chloroform at 4°C.

### Transmission electron microscopy

Isolated phage solution (5 μl) was collected for morphological characterization *via* negative staining. The samples were stained with 0.5% (w/v) aqueous uranyl acetate (Chopin et al., 2002) and visualized by transmission electron microscopy (TEM; Technai Bio TWIN) at 80 kV with a magnification of 40,000–100,000×.

### DNA extraction and sequencing

Phages were concentrated by centrifugation at 30,000 rpm (JA-30.50 fixed angle rotor, Beckman Coulter) for 2 h at 4°C. The supernatant was carefully removed and phages were re-suspended in 200 μl SM-Buffer. TURBO DNase™ buffer (20 μl) and TURBOTM DNase (2 μl) (Invitrogen, Thermo Scientific) were added and incubated at 37°C for 1 h. Afterward, we added 22 μl of 2 M Tris–HCl (pH 8.5, 0.2 M EDTA, 20 μl 10% SDS, 10 μl 0.5 M EDTA) and 6 μl Proteinase K (ROTH) to each sample and incubated at 37°C for 20 min and 56°C for 15 min. We added 800 μl of CTAB Extraction Buffer (100 mM Tris at a pH of 8, 3 M NaCl, 20 mM EDTA, and 3% Cetyltrimethylammonium bromide) to all samples and incubated at 65°C for 10 min. Samples were purified twice by adding Chloroform:Isoamyl alcohol 24:1 (MERCK) in a 1:1 ratio. After centrifugation at 13,000 ×g for 5 min, the supernatant was transferred into a new tube and DNA was precipitated by adding 0.7× volume isopropanol. The samples were stored at −20°C overnight and centrifuged at 13,000 ×g, 4°C for 20 min. DNA pellets were washed with 70% ethanol (v/v) and re-suspended in 40 μl DNase-free water for sequencing. Nextera XT kit (Illumina) was used for library preparation and phage DNA was 2 × 150 bp paired-end sequenced on a MiSeq platform (Illumina) at the IKMB in Kiel. Adapters were removed and reads were trimmed by Trimmomatic V.0.36 (Bolger et al., 2014). The phage genome was finally assembled using SPAdes V.3.1.14 (Bankevich et al., 2012). The assembled *Curvibacter* phage PCA1 genome will be publicly available under GenBank accession number: BankIt2629455 Seq1 OP588919.

### PCA1 phage genome annotation

Open reading frame (ORF) prediction and functional annotation of PCA1 phage was done using a combination of different gene finders; PHANOTATE (McNair et al., 2019) Pharokka (Steinegger and Söding, 2017), Prodigal (Hyatt et al., 2010), Prokka (Seemann, 2014), Bakta (Aziz et al., 2008), GeneMarkS (Besemer et al., 2001) RAST (Aziz et al., 2008), and Balrog (Sommer and Salzberg, 2021). Consensus gene calls and best hit predicted protein similarity searches were made using PHROGs (Terzian et al., 2021), VOG,^1^ eggNOG (Huerta-Cepas et al., 2019), PFAM (Mistry et al., 2021), PhaLP (Criel et al., 2021), and ACLAME (Leplae et al., 2010). Databases were curated manually. Putative transfer RNA (tRNA) genes were identified using ARAGORN (Laslett and Canback, 2004) and tRNAScan-SE (Schattner et al., 2005). The graphical genome map was generated with the CGView server tool (Stothard et al., 2019) and grouped by PHROGs functional categories. The classification into head, neck, and tail proteins of tailed bacteriophages was done with VIRFAM (Lopes et al., 2014). The phylogenetic placement and viral proteomic tree construction with closely related phage genomes were done with VipTree (Nishimura et al., 2017).

### Host range assay

Phage solution was spotted on top of several overlay agar plates (see above) containing different bacterial species, including *Curvibacter* sp. AEP1.3, *Duganella sp.* C1.2, *Acidovorax sp.* AEP1.4, *Pelomonas sp.* C7.1, *Pelomonas sp.* AEP2.2, *Pseudomonas sp.* T1, *Pseudomonas sp.* T3, *Exiguobacterium sp.* C4.1, *Oxalobacteraceae sp.* C1.1, *Chryseobacterium sp.* C3.1, *Curvibacter sp.* P1.1, *Pseudomonas anguilliseptica* AEP1.1, *Vogesella* sp. AEP1.1, *Curvibacter sp.* Mag1.1, *Curvibacter sp.* Hvul, *Duganella sp.* Oli1.1, *Pseudomonas sp.* Oli1.2, *Flavobacteriales sp.* T2, and *Chryseobacterium sp.*. The plates were incubated at RT for 4 days and observed for plaque formation every 24 h.

### Phage infection in liquid bacterial culture

96-well plates (CELLSTAR®, Greiner bio-one) were loaded with 200 μl sample solution, consisting of sterile R2A medium for negative controls and positive controls containing 200 μl liquid *Curvibacter* sp. AEP1.3 culture in R2A at a starting OD of 0.2. phage solution (10 μl) (10:1 phages to bacterial cells) was added to each sample containing 180 μl of bacterial culture (n = 5). Bacterial growth was measured at 20°C and 600 nm OD using a plate reader (Spark®, TECAN). OD measurements were taken every 15 min over the course of 24 h, with orbital shaking at 150 rpm preceding each interval.

### Testing for resistance development

*Curvibacter* sp. AEP1.3 previously exposed to PCA1 phage in liquid culture, without showing a decline in bacterial growth after 24 h, were mixed into overlay agar and tested for their susceptibility, by spotting PCA1 phage on top of an overlay agar containing previously exposed bacteria (see above).

### Localization of attached and unattached phages

Subsamples (100 μl) of liquid *Curvibacter sp.* AEP1.3 culture infected with PCA1 were taken after 48 h of incubation (n = 5) and diluted in series. Dilution (100 μl) 1, 1/100, and 1/100.000 was added to 900 μl Curvibacter (OD 0.2) and mixed into 4 ml overlay agar. The same was done with dilutions that were treated with Chloroform.

### Impact of surface area on phage infection

*Curvibacter* sp. AEP 1.3 at 0.2 OD_600_ were exposed to 23,000 PFU/ml PCA1 phage solution. bacterial-phage mixture (5 ml) was transferred into 10 glass vials. Five glass vials were filled with 0.6 g glass wool to increase the surface area and 5 without glass wool served as controls. After 24 h PFU were quantified.

### Small molecule extraction

We cultured *Curvibacter* sp. AEP1.3 on 10 R2A agar plates at 18°C for 48 h, rinsed off bacterial cells with liquid R2A, and transferred them into 2 ml Eppendorf tubes. Simultaneously, we transferred *Curvibacter* sp. AEP1.3 from liquid culture into 2 ml tubes and adjusted OD to match that of plated bacteria. The tubes were placed on ice and bacterial cells were lysed using a sonicator (BANDELIN electronic, 2,200). The lysate was transferred into 50,000 Molecular weight cut-off (MWCO) cartridges (Sartorius Stedim Biotech, VIVASPIN TURBO 15) and centrifuged at 4,600 rpm for 15 min (SORVALL Heraeus fixed angle rotor 75,006,445). Both flow-through (small fraction) and the residue (large fraction) were transferred into new 1.5 ml tubes. Flow-through (50 μl) was added to 150 μl of liquid *Curvibacter* sp. AEP1.3 culture and placed into a 96-well plate. PCA1 phage (10 μl) was added to the mixture. Simultaneously, 50 μl of large fraction were added to 150 μl of liquid *Curvibacter sp.* AEP1.3 culture in another well together with 10 μl of PCA1 phage. Non-treated *Curvibacter sp.* AEP1.3 culture and AEP1.3 with PCA1 phage served as controls (*n* = 4). The growth of all cultures was measured at 600 nm OD using a plate reader (Spark® TECAN). Conditions were set to 18°C and shaking (150 rpm) before each measurement. OD was measured every 15 min.

### Modification of fluorescent *Curvibacter sp.* AEP1.3

For *in vivo* visualization of *Curvibacter sp.* AEP1.3, we chromosomally integrated a dTOM *via* the miniTn7 system as previously described (Wiles et al., 2018). The protocol was modified and adjusted to *Curvibacter sp.* AEP1.3 in the following way: Instead of E.coli strain SM10 we used MFDpir, because triparental mating was already established for *Curvibacter sp*. (Wein et al., 2018). We used R2A for plates and media with the exception of pure *E. coli* cultures. For reaching a 1:1:1-ratio during mating, we used 10 ml overnight grown (30°C) *Curvibacter sp*. AEP1.3 culture (OD600 ≤ 0.3), pelleted it (4,000 ×g), and re-suspended it in 0.5 ml. In addition, the concentration of Gentamicin in selection plates was reduced to 0.2 μg/ml as described (Wein et al., 2018). All other steps were performed as described by Wiles et al. (2018).

### Phage treatment of mono-colonized *Hydra vulgaris*

*Hydra vulgaris* AEP polyps were kept in S-Medium (0.042 g/l CaCl_2_, 0.8 g/l MgSO_4_, 0.4 g/l NaHCO_3,_ and 0.1 g K₂CO_3_) in ultrapure, autoclaved water (Lenhoff and Brown, 1970) at 18°C. Polyps were fed bi-weekly with *Artemia salina* shrimp (Sanders®, Great Salt Lake Artemia) so that each polyp received 3 to 5 shrimps. *A. salina* were imported as cysts and hatched in salt water (Loomis, 1953). Polyps were washed 6 to 12 h after feeding. To create germ-free (GF) *Hydra vulgaris* polyps, we transferred 25 wild-type (Wt) polyps into a sterile cup containing 50 ml of S-Medium and antibiotic solutions [50 mg/ml of: Ampicillin, Rifampicin, Spectinomycin, Streptomycin, and Neomycin (Franzenburg et al., 2012)]. The polyps in antibiotic medium were stored at 18°C and kept in the dark. Over the course of 2 weeks, medium was exchanged with new S-Medium and the same quantity of antibiotics every third day. Afterward, polyps were placed in S-medium without antibiotics for 2 days before exchanging the medium once more with fresh S-Medium. We colonized 20 of the GF-Hydra by transferring them into a separate cup and adding 50 μl of 0.2 OD *Curvibacter* sp. AEP1.3 RFP. The cups were incubated at 18°C for at least 48 h, until a fluorescent signal became visible on mono-colonized *Hydra* under light microscopy. We removed both GF and mono-colonized polyps from their 50 ml cups, placed each polyp into a 1.5 ml tube (Eppendorf), and filled it with 1 ml fresh S-Medium. Five mono-colonized polyps were treated with 20 μl PCA1 phage solution (10:1 phages to bacterial cells), five with heat-inactivated PCA1 phage solution, and five mono-colonized polyps were left untreated (*n* = 5). In order to add phage solution, 2 ml of the phage stock (see above) was placed into a 50,000 molecular weight cut-off Vivaspin® cartridge, centrifuged at 4,000 g for 25 min, and washed with S-medium. Phages were retained and re-suspended in S-Medium while the flow-through containing R2A was discarded. Heat inactivation was achieved by boiling 3 ml of phage solution at 95°C for 15 min. After 24 h of incubation at 18°C, polyps were washed twice in sterile S-medium and homogenized in 200 μl S-Medium. Pestles were rinsed off with an additional 800 μl of S-Medium. We plated out 100 μl of homogenate onto R2A agar plates, incubated them at RT for 48 h, and counted colony-forming units (CFU).

### Statistical analysis

We analyzed and visualized our data using GraphPad Prism 9.3.1. Bacterial growth was plotted in a linear XY function, with error bars indicating standard deviation. For CFU and PFU count analysis, Box–Whisker plots were created using the 5 to 95 percentile. Significance of results was determined using a Shapiro–Wilk test to determine if data followed a normal distribution. Additionally, we performed one-way ANOVA with Tukey’s multiple comparisons test. Some data did not pass the normality test (α = 0.05) but ANOVA is relatively robust against violation of the assumption of homoscedasticity (Underwood, 1997). Data were analyzed parametrically at a lowered a-level of 0.01 (Wakefield and Murray, 1998) to counteract the increased probability of false positives.

### RNA extraction

*Curvibacter* sp. AEP1.3 were grown to 0.2 OD and distributed onto solid R2A agar plates using glass beads. After 48 h of incubation at RT, we harvested the bacterial lawns by adding 100 μl of sterile ultrapure water and scraping the mixture into a 2 ml tube. Simultaneously, *Curvibacter* cells were pelleted from fresh 0.2 OD liquid culture. For collecting samples of *Curvibacter sp.* mono-colonizing *Hydra vulgaris* AEP, we collected 500 polyps for one replicate and washed off bacteria by using 1x PBS solution as described previously and pelleted the cells (Pietschke et al., 2017). Bacterial samples from all three treatments were dissolved in 750 μl trizol and frozen overnight at −80°C. Chloroform (250 μl) was added to each sample, which were mixed and incubated at RT for 5 min. The samples were centrifuged at 12,000 g for 15 min at 4°C, after which the upper phase was mixed with 1× volume of ethanol and transferred into Spin Cartridges. Afterward, we followed instructions according to the PureLink™ RNA Mini Kit (Thermo Fisher Scientific) with the exception that we doubled all washing steps. RNA was eluted into 35 μl RNase free water and stored at −80°C until samples were collected.

### Sequencing and analysis

Isolated RNA was prepared using the TruSeq stranded total RNA kit (Illumina) and Ribo-Zero Plus kit (Illumina) according to protocol. The remaining RNA was paired-end sequenced by a NovaSeq 6000 (Illumina) with 2 × 150 bp. RNA sequences were analyzed according to Batut et al. (2018, 2022). As such, we used Cutadapt (Martin, 2011), Trimmomatic (Bolger et al., 2014), FastQC (Andrews, n.d.), and MultiQC (Ewels et al., 2016) for quality control. We used Bowtie2 (Langmead et al., 2009) to map our reads to the *Curvibacter* genome (Pietschke et al., 2017) and featureCounts (Liao et al., 2013) to count reads. Finally, we used Deseq2 (Love et al., 2014) for differential gene expression analysis and GraphPad Prism version 9.4.0 for graphical representation. Our raw RNA sequencing data are publicly available at the sequencing read archive database under the Bioproject accession number PRJNA887579.

## Results

### Isolation and classification of *Curvibacter* phage PCA1

**Figure 1:**
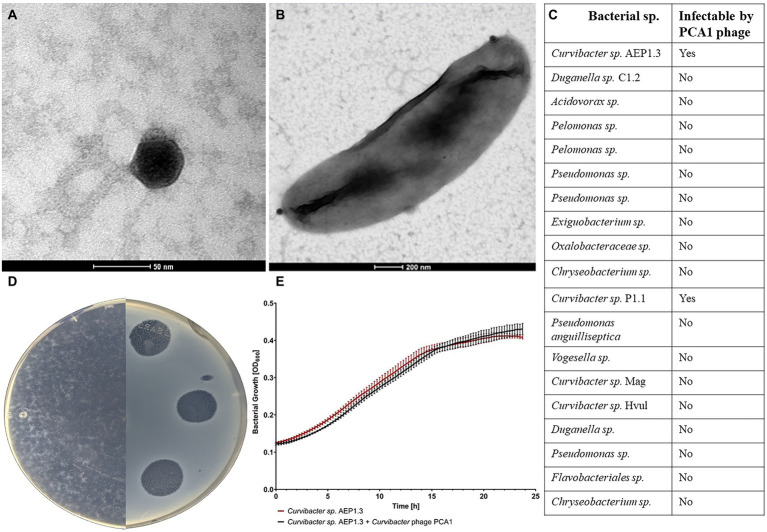
**(A)** TEM image of *Curvibacter* phage PCA1 stained with uranyl acetate. **(B)** TEM image of *Curvibacter* sp. AEP1.3 cell infected with *Curvibacter* phage PCA1. **(C)** Table depicting host range of *Curvibacter* phage PCA1 with bacterial strains in the first column and ability to infect in the second. **(D)** Image of R2A agar plates containing a bacterial lawn of *Curvibacter sp.* AEP1.3 infected with *Curvibacter* phage PCA1. **(E)** Bacterial growth of *Curvibacter sp.* AEP1.3, with and without PCA1 phage, in liquid R2A medium. Recorded *via* Optical Density measurements at 600 nm for 24 h (*n* = 5).

After isolating *Curvibacter* phage PCA1 from nutrient-enriched freshwater collected from the Bioturm pond, we used TEM to obtain visual confirmation of phage presence and to classify PCA1 according to morphology. PCA1 phage possessed an icosahedral head with a diameter of 50 nm and an approximately 150 nm long, non-contractile tail, giving it a total length of approximately 200 nm (Figure 1A). This indicated that PCA1 belonged to the order of *Caudovirales* and the *Siphoviridae* family (Tolstoy et al., 2018). This finding was confirmed by taxonomic placement in proteomic tree analysis, where PCA1 phage clustered together with reference *Siphoviridae* phages from other Proteobacteria. The phage was placed on its own branch due to overall low genomic similarity however (<50%; Supplementary Figure S1). Observation under TEM additionally allowed us to observe attachment to a *Curvibacter* sp. AEP1.3 cell. PCA1 phages attached to the bacterial surface such that only dark, icosahedral heads were visible at the bacterial cell poles (Figure 1B).

We then proceeded with spot assays in order to gauge PCA1’s host range. Testing against multiple available bacterial strains, including other isolates from *Hydra vulgaris* AEP such as *Duganella sp.* and *Acidovorax sp.*, showed that PCA1 only infected *Curvibacter sp.* AEP1.3 and *Curvibacter sp.* P1.1 (Figure 1C). One such spot assay is depicted in Figure 1D, where we infected *Curvibacter sp.* AEP1.3 with PCA1 phage solution on an R2A agar plate. Areas of infection were transparent in contrast to the surrounding opaque bacterial lawn. Notably, PCA1 phage plaques continued to expand in size several days after initial infection until they eradicated the entire surrounding bacterial lawn. This occurred at RT as well as at lower temperatures down to 8°C, at a 10:1 ratio of phages to bacteria and lower (Figure 1D).

### Selective infectivity of PCA1 phage

During amplification of PCA1 for downstream analysis such as DNA extraction, we followed standard procedure by infecting increasing amounts of *Curvibacter* sp. in liquid culture, only to find that phage concentration did not seem to increase. In order to investigate this observation, we tested for phage infectivity by measuring optical density (OD) at 600 nm as an indicator of bacterial growth. Usually, one would see exponential growth in non-infected bacteria, while phage infection would result in a sudden OD drop due to cell lysis. In this case, however, we observed that *Curvibacter sp.* AEP1.3 grew exponentially to an OD of 0.4 within 20 h, regardless of phage presence or absence. In conclusion, phage infection and cell lysis were observed on solid R2A agar but did not occur in liquid culture containing *Curvibacter sp.* AEP1.3 (Figure 1E).

Due to this circumstance, we collected PCA1 phage DNA for extraction by amplifying phages on R2A agar plates instead of liquid culture. Resulting genomic analysis showed that the PCA1 phage genome consisted of 57,776 bp dsDNA, and contained 84 protein-coding genes and one tRNA gene.

**Figure 2:**
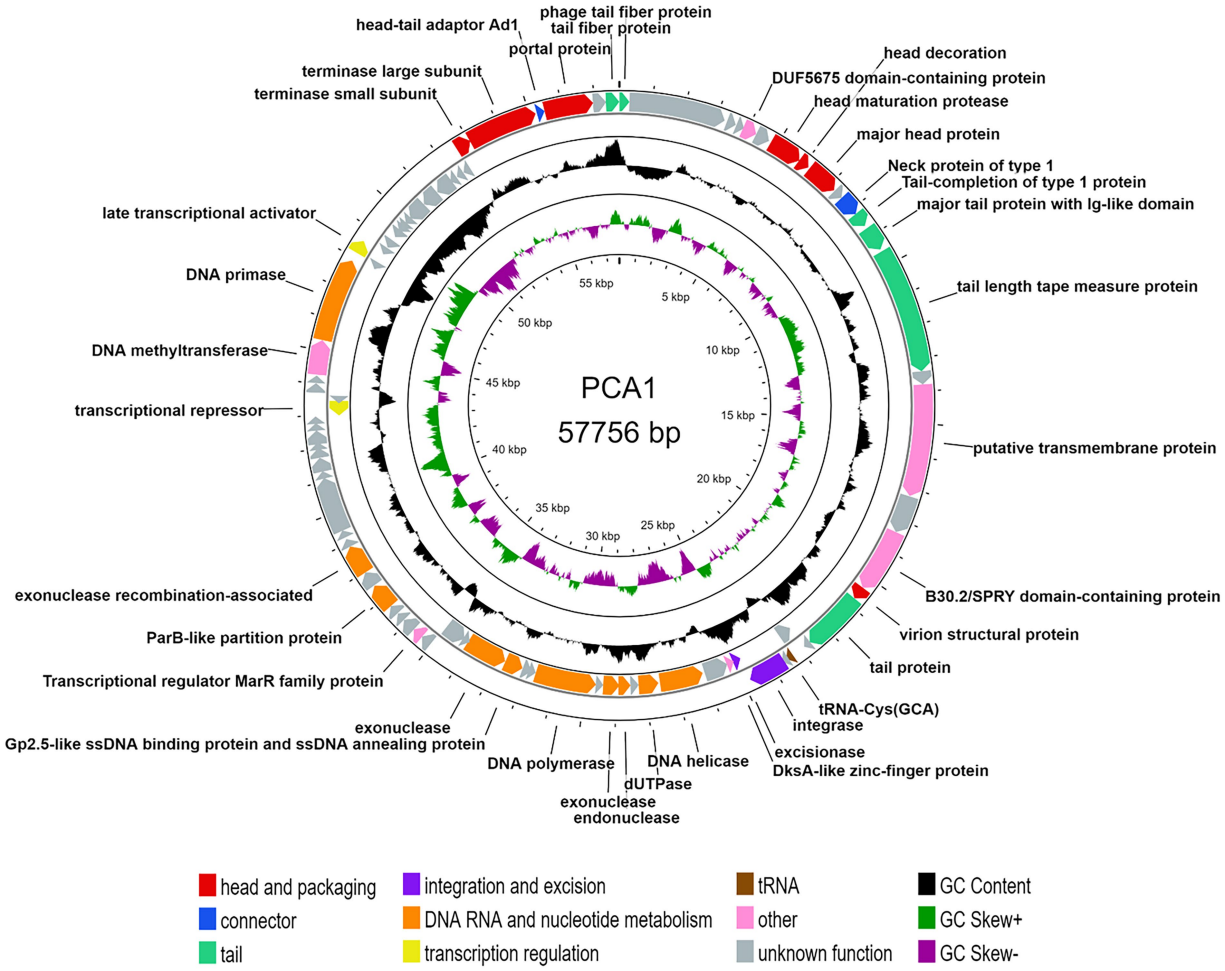
Circular genomic map of bacteriophage PCA1. The two outer rings show 84 ORFs and one tRNA gene, based on the direction of transcription and colored by their different functional categories: head and packaging (red), connector (blue), tail (teal), integration and excision (purple), DNA RNA and nucleotide metabolism (orange), transcription regulation (yellow) and other proteins (pink), tRNAs (brown), and hypothetical proteins (gray). The two inner rings with histogram bars show GC content (black), positive GC skew (green), and negative GC skew (violet).

35 (41.66%) of all ORFs could be assigned with putative function while the rest were assigned as hypothetical proteins, out of which at least 12 matched other phage genomes and predicted genes with unknown function. Alongside standard phage proteins, such as head and tail proteins, the PCA1 phage boasted a large set of transcription machinery including DNA polymerases and helicases, as well as nucleases and transcriptional activators. Last but not least, presence of an integrase indicated that the PCA1 phage was a temperate phage (Figure 2).

Since phage presence was confirmed, bacterial resistance remained as another potential cause for the observed lack in phage infectivity in liquid culture. To test this, we mixed liquid *Curvibacter* sp. AEP1.3 culture previously exposed to PCA1 phage into overlay agar and additionally spotted PCA1 phage on top. After 2 days of incubation at RT, plaques not only became visible in the areas where we had spotted phages, but all over the agar plate (Supplementary Figure S2A). This observation suggested that PCA1 phage persisted in liquid culture without causing lytic infection.

To test whether PCA1 phage coexisted in liquid culture without attaching or if the PCA1 phage infected bacterial cells without lysis due to prolonged eclipse time, we quantified phage attachment in liquid culture over time. PFU counts of samples treated with chloroform (used to remove infected and uninfected bacterial cells, only leaving unattached phages in the medium), showed that PCA1 phages were not attached to bacterial cells and persisted as free phages in liquid cultures.

### Inducing PCA1 phage infection in liquid culture

Subsequently, we tested multiple variables to try and induce phage infection in liquid culture. Since *Curvibacter* sp. AEP1.3 in liquid culture were exposed to orbital shaking, we hypothesized that this movement could interfere with phage attachment. Cessation of shaking did not result in phage infection of liquid *Curvibacter* sp. AEP1.3. Secondly, we added R2A agar to our liquid culture, so that nutrient conditions would be the exact same. This did not induce phage infection. Since *Curvibacter* are exposed to a brief heat shock when added to overlay agar, we mimicked this by briefly increasing temperature in liquid cultures, which did not induce infection either. In order to improve phage attachment, we added Mg^2+^ and Ca^2+^ cations to liquid *Curvibacter* sp. AEP1.3 culture. This did not induce phage infection as well (Supplementary Figure S2B).

*Curvibacter* express 3-oxo-homoserine lactones on solid medium, among which 3-OHC_12_-HSL has been correlated with biofilm formation, resulting in our hypothesis that it may make *Curvibacter* susceptible to infection, but addition of *Curvibacter* homoserine lactones did not induce phage infection (Supplementary Figure S2C).

**Figure 3:**
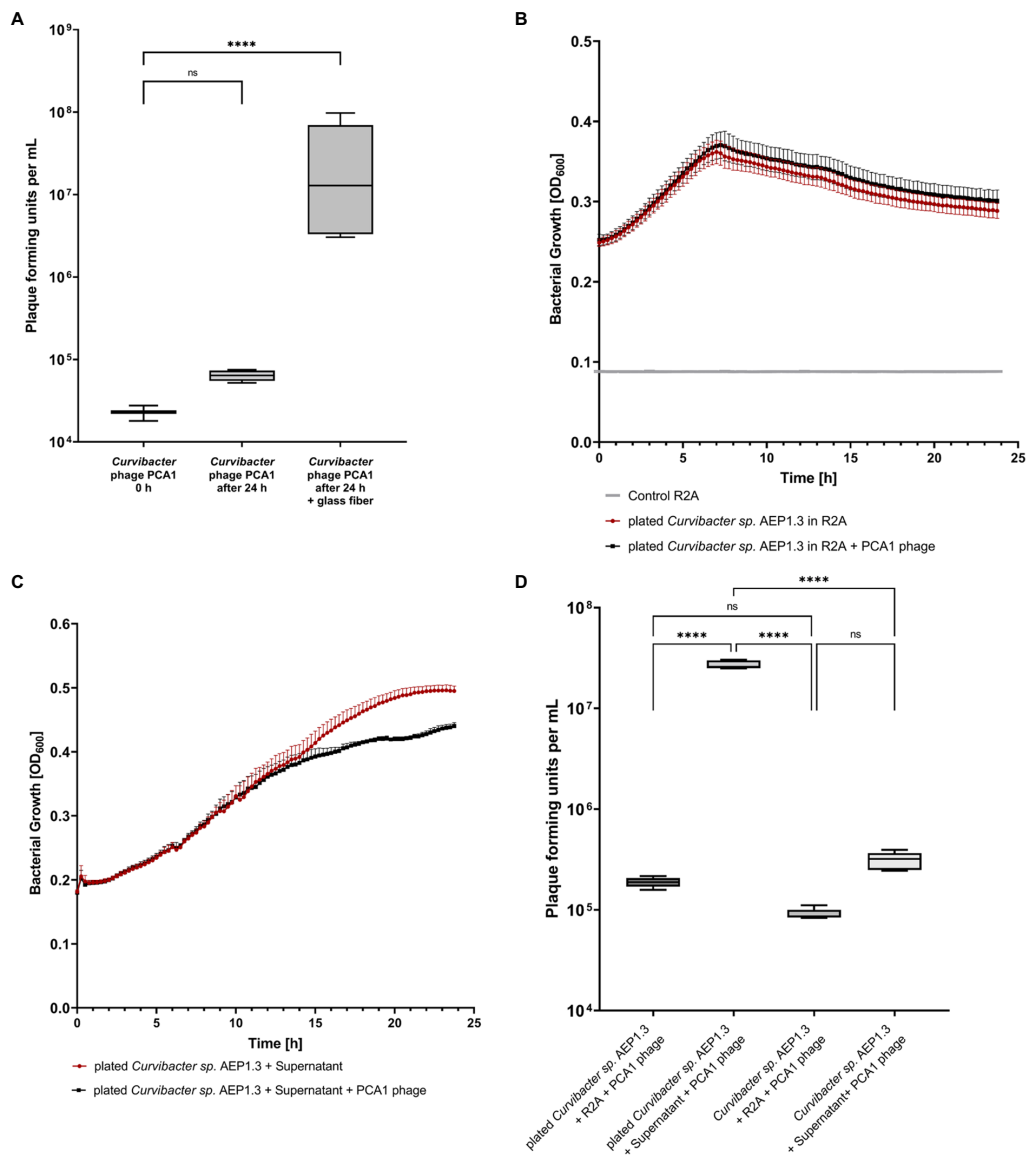
**(A)** Box-Plot diagram displaying plaque forming unit counts of *Curvibacter* phage PCA1 in liquid culture and liquid culture filled with glass fiber. **(B)** Growth curves of *Curvibacter sp.* AEP1.3 with and without *Curvibacter* phage PCA1. Bacteria were grown on solid medium and transferred into liquid culture, where growth was determined *via* optical density (OD) at 600 nm (*n* = 5). **(C)** Growth curves showing *Curvibacter sp.* AEP1.3 transferred from solid medium to liquid culture, measured at OD_600_. Supernatant from plated *Curvibacter sp.* AEP1.3 was added to cultures alongside PCA1 phage. (*n* = 4). **(D)** Plaque forming unit counts of *Curvibacter* phage PCA1 in *Curvibacter sp.* AEP1.3 cultures. Treatments included addition of supernatant or R2A to bacteria in liquid culture or bacteria transferred from solid agar plates to liquid culture (*n* = 5). “^****^” indicates adjusted *p*-value beneath 0.0001 in Tukey’s multiple comparisons test.

Finally, we added glass fiber to liquid *Curvibacter* sp. AEP1.3 culture, in order to provide a surface for bacterial cell adhesion similar to solid medium. This procedure succeeded in increasing PCA1 infectivity. When adding 10^4^ PCA1 PFU to *Curvibacter* sp. AEP1.3 in liquid culture, the amount of phages increased non-significantly to 10^5^ after 24 h of incubation, while the addition of glass fibers resulted in a significant increase to 10^7^ (Figure 3A).

Since substrate texture had been shown to have an effect on phage infectivity, we hypothesized that adhesion proteins in the bacterial membrane could be responsible for phage infectivity. These proteins should remain stable within the membrane for several minutes, so that bacterial cells transferred from solid medium to liquid culture should still be susceptible to infection. We proceeded to investigate this hypothesis by transferring *Curvibacter* sp. AEP1.3 from R2A agar plates into liquid culture. OD_600_ measurements conducted with *Curvibacter* sp. AEP1.3 and *Curvibacter sp.* AEP1.3 with PCA1 phage showed that bacterial growth remained the same. Thus, PCA1 phages were not able to infect transferred *Curvibacter* sp. AEP1.3 either (Figure 3B).

This outcome changed when we repeated the experiment with the addition of supernatant from *Curvibacter sp.* AEP1.3 plated on solid agar to *Curvibacter* sp. AEP1.3 cells cultured on solid agar. While growth curves in liquid culture looked similar to previous experiments for the first 12 h, bacterial growth of cultures containing PCA1 phage stagnated after 13 h at 0.38 OD_600_, whereas *Curvibacter* sp. AEP1.3 with supernatant and no PCA1 phage grew exponentially until an OD of 0.5 (Figure 3C). After splitting supernatant derived from plated *Curvibacter* sp. into fractions above and below 10,000 MWCO, both addition of the larger fraction (LF) and small fraction (SF) supernatant resulted in a decrease in bacterial growth on previously plated *Curvibacter* sp. AEP1.3 after 13 h. The addition of R2A as a negative control instead of supernatant did not result in a decrease in bacterial growth, despite addition of PCA1 phage (Supplementary Figure S2E).

In order to confirm that the observed decrease in bacterial growth was indeed caused by phage lysis and not an adverse reaction to the supernatant, we quantified the amount of PCA1 phages present. Adding 10^5^ PCA1 phages to *Curvibacter* sp. AEP1.3 derived from liquid culture did not yield a significant increase in phage concentration after 24 h of incubation, even when supernatant from plated bacteria was added. Adding PCA1 phages to *Curvibacter* sp. AEP1.3 transferred from solid agar into liquid culture did not result in phage amplification either but addition of supernatant to previously plated *Curvibacter* sp. AEP1.3 resulted in a significant increase of PCA1 phage from 10^5^ PFU to 10^8^ PFU (Figure 3D).

### PCA1 infectivity on *Hydra vulgaris* AEP

**Figure 4:**
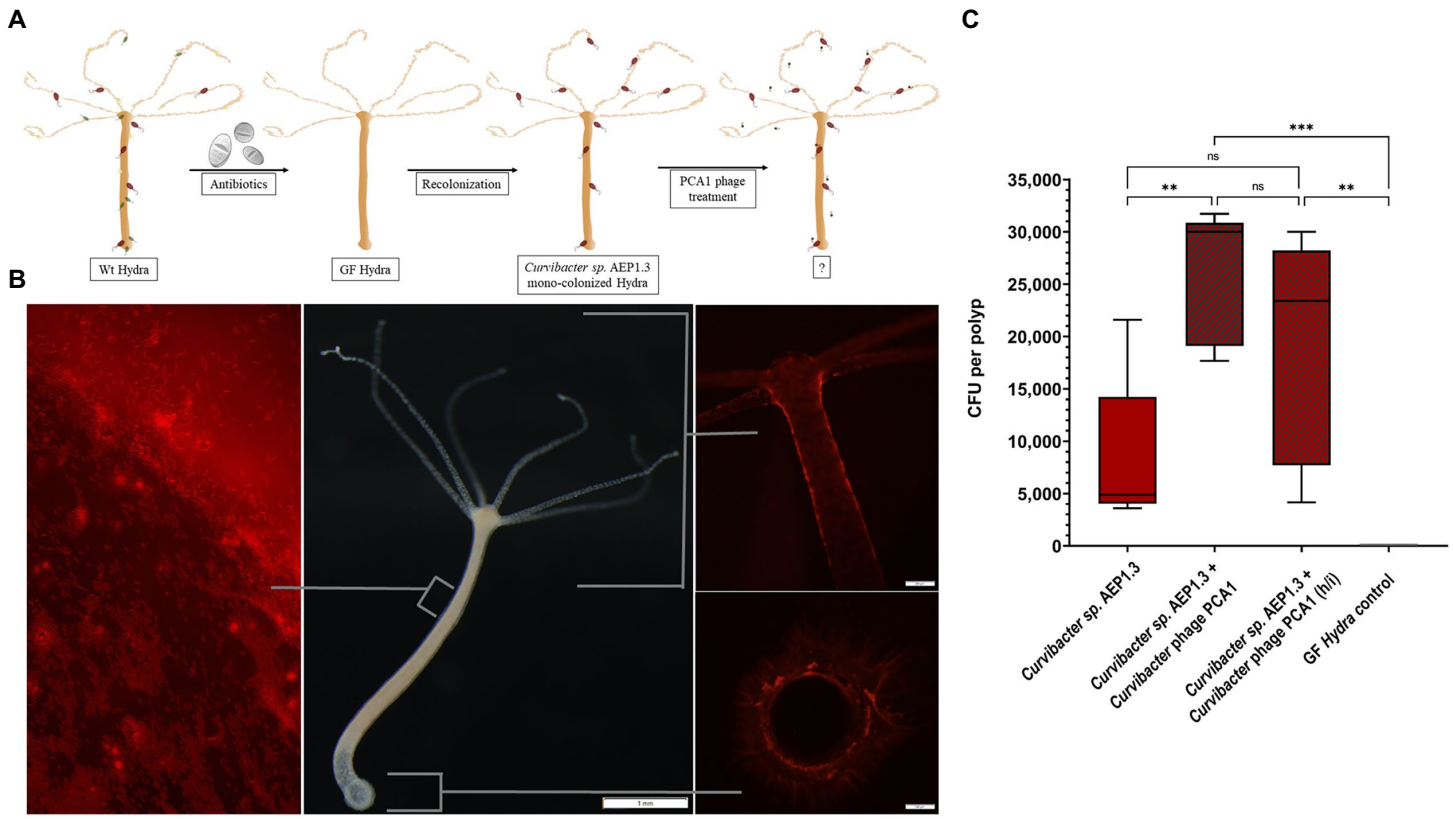
**(A)** Schematic showing experimental setup, starting with antibiotic treatment of wild-type *Hydra vulgaris AEP*. Resulting germ-free *Hydra vulgaris AEP* were recolonized with *Curvibacter sp.* AEP1.3. The mono-colonized *Hydra* were then exposed to *Curvibacter* phage *PCA1.* **(B)** *Hydra vulgaris AEP* mono-colonized with fluorescently labeled *Curvibacter sp.* AEP1.3. The left image shows a section of the body column and mucus layer under fluorescent light, the middle image shows a *Hydra vulgaris* AEP polyp under white light. The image to the right shows the head and foot region under fluorescent light. **(C)** Colony-forming unit counts of *Curvibacter sp.* AEP1.3 mono-colonized polyps with and without *Curvibacter* phage PCA1 and a germ-free control (*n* = 5). Heat-inactivated phages are indicated by “h/i.” ** indicates adjusted *p*<0.0077, ns indicates non-significant data, *** indicates *p* = 0.0002.

While we learned that *Curvibacter* sp. AEP1.3 were susceptible to PCA1 infection on solid medium but not in liquid culture, our initial goal was to selectively remove *Curvibacter* sp. AEP1.3 from the microbiota of *Hydra vulgaris* AEP. Thus, the question remained whether *Curvibacter* sp. AEP1.3 could be infected by PCA1 phage when associated with its host. This question could also be used to infer whether the behavior of *Curvibacter sp.* AEP1.3 on *Hydra* resembled that of sessile bacteria on solid medium or that of planktonic bacteria in liquid culture. In response, we removed bacteria from wild-type (Wt) *Hydra vulgaris* AEP using antibiotics, so that polyps could be colonized with *Curvibacter* sp. AEP1.3 and exposed mono-colonized polyps to PCA1 phage (Figure 4A).

To quantify the amount of *Curvibacter* sp. AEP1.3 on *Hydra* before and after PCA1 phage treatment, we labeled *Curvibacter sp.* AEP1.3 with red fluorescent protein (RFP), which allowed us to observe presence, absence, and colonization patterns of RFP labeled *Curvibacter sp.* AEP1.3 on *Hydra vulgaris* AEP. Fluorescent signals could be observed on tentacles, around the body column, and basal disk but not within *Hydra.* The majority of signals were located in the mucus layer surrounding *Hydra,* where single rod-shaped signals could be observed, indicating that *Curvibacter sp.* AEP1.3 RFP swam freely within the mucus layer (Figure 4B).

Adding PCA1 phage to mono-colonized *Hydra* polyps did not result in a visible reduction of fluorescence. This observation was further supported by CFU counts of homogenized polyps. While germ-free polyps did not contain any bacteria, *Curvibacter sp.* AEP1.3 mono-colonized polyps housed an average of 5,000 CFU. Mono-colonized polyps treated with PCA1 phage contained even more *Curvibacter sp.* AEP1.3 with an average of 30,000 CFU while polyps treated with heat-inactivated PCA1 phages housed an average count of 23,000 CFU (Figure 4C). In summary, PCA1 phage did not eliminate the fluorescent signal derived from RFP labeled *Curvibacter* sp. AEP1.3 on mono-colonized *Hydra* nor did it reduce the amount of colony-forming units per polyp.

### Adaptive lifestyle of *Curvibacter sp.* AEP1.3

So far this study indicated clear differences when comparing *Curvibacter* sp. AEP1.3’s susceptibility to PCA1 phage infection (Figures 1D,E, 4C), such that *Curvibacter sp.* could be divided into two groups; one consisted of phage-immune *Curvibacter sp.* AEP1.3 in liquid culture and on *Hydra*, while the other group consisted of susceptible *Curvibacter* sp. AEP1.3 on solid medium. Since *Curvibacter sp.* AEP1.3 in liquid culture and on *Hydra* were both immune to phage infection, we hypothesized that *Curvibacter* on *Hydra* are in the same state as *Curvibacter* in liquid culture.

**Figure 5:**
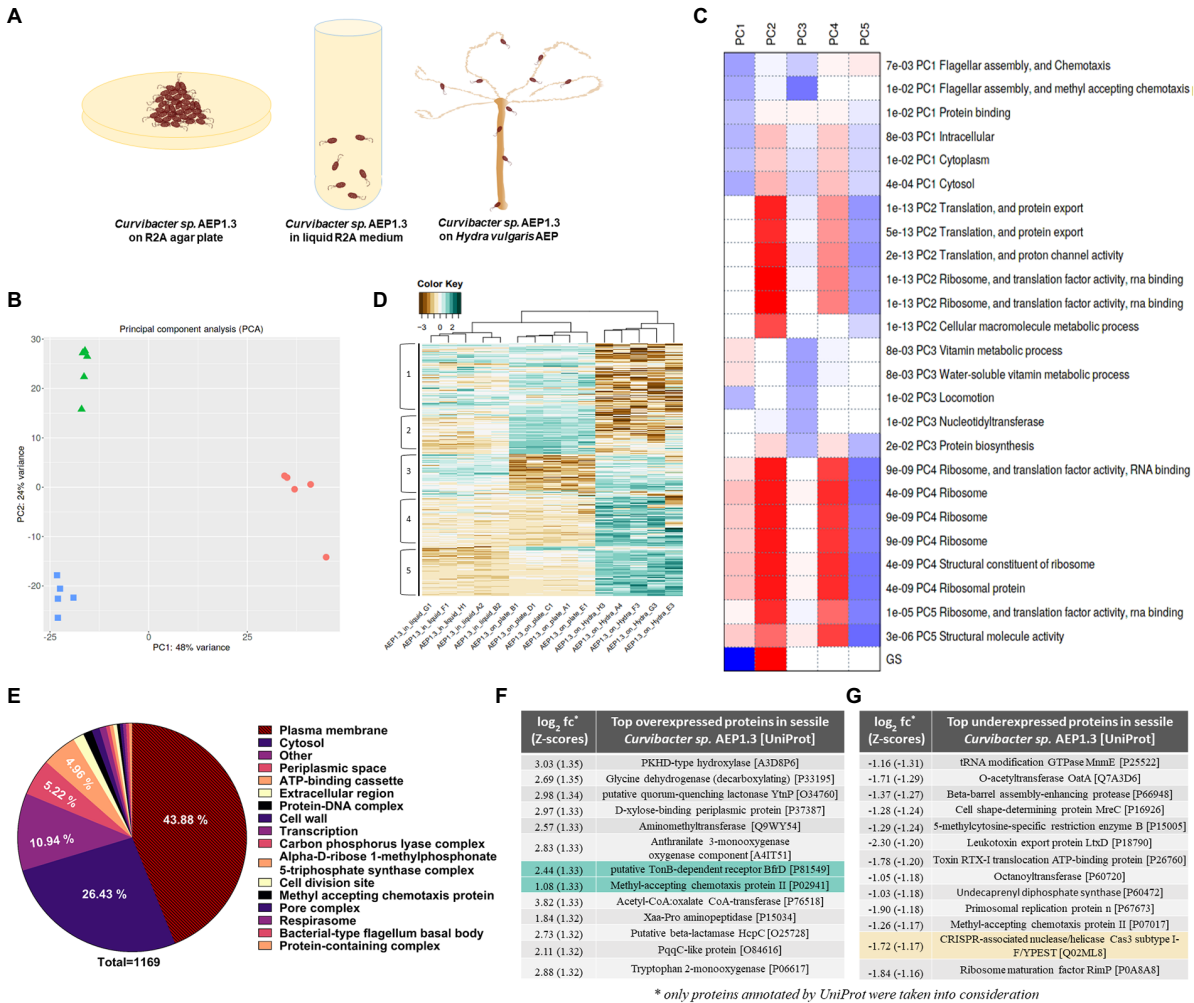
**(A)** Hypothetical model of sessile *Curvibacter sp.* AEP1.3 on solid R2A agar (left), planktonic *Curvibacter sp.* AEP1.3 in liquid R2A medium (middle) and *Curvibacter sp.* AEP1.3 on their host *Hydra vulgaris* AEP (right). **(B)** Principal component analysis comparing RNA sequencing results of *Curvibacter sp.* AEP1.3 on solid medium, liquid medium, and on *Hydra n* = 5. **(C)** Table showing factors contributing to principal components 1–5, based on molecular function. **(D)** Clustered heatmap based on log^2^ + 1 transformed Z-scores to visualize differences in expression patterns between sessile, planktonic, and host-associated *Curvibacter sp.* AEP1.3 with scores ranging from −3 to 2. **(E)** Pie diagram showing the percentage of proteins differentially expressed in *Curvibacter sp.* AEP1.3 on solid medium in comparison to planktonic and host-associated bacteria. Proteins were classified using the UniProt database. **(F)** Top proteins upregulated in sessile *Curvibacter sp.* AEP1.3 in comparison to planktonic and host-associated *Curvibacter,* sorted by Z-scores of log^2^ fold changes. Green highlights indicate potential candidates for PCA1 phage binding. **(G)** Top proteins downregulated in sessile *Curvibacter sp.* AEP1.3, sorted by Z-scores of log^2^ fold changes. Yellow highlights indicate potential candidates for defense against PCA1 phage infection.

In order to test this hypothesis, gain deeper insight into bacterial expression patterns and find potential phage-binding candidates, we compared sessile *Curvibacter* sp. AEP1.3 on solid medium, planktonic *Curvibacter* in liquid culture, and host-associated *Curvibacter* on Hydra (Figure 5A) *via* RNA sequencing.

Conducting principal component analysis (PCA) on transcriptome samples derived from sessile, planktonic, and host-associated *Curvibacter sp.* AEP1.3 showed that they clustered closely together within their treatment group and differed strongly between treatments. Axis one, with a variance of 48%, separates *Curvibacter* on Hydra from *Curvibacter* in liquid culture and on plate. Axis two (24% variance) separates *Curvibacter* in liquid culture from those on plate. Overall, the three treatments formed a triangle with an overall PC1 variance of 48% and 24% PC2 variance (Figure 5B).

The downregulation of flagellar assembly proteins and chemotaxis proteins, as well as protein-binding components and intracellular processes, were the main contributors to principal component 1. Principal component 2 was defined by upregulated proteins involved in translation, including RNA-binding and ribosomal proteins, while principal component 3 was defined by downregulation of vitamin metabolism and cell movement. Principal component 4 was similar to component 2, as it was mostly affected by processes involved in translation, whereas these same processes were downregulated for PC5 (Figure 5C).

Differences between sessile, planktonic, and host-associated *Curvibacter* sp. AEP1.3 remained visible when looking at overall gene expression, since samples clustered together accordingly. *Curvibacter* sp. AEP1.3 in liquid culture showed expression levels between Z-scores (#standard-deviations difference from mean) of 0 to 1 in areas 1 and 3, while expression in other areas ranged between Z-scores of −1 and − 2. *Curvibacter sp.* AEP1.3 on plates showed high expression levels in areas 1 and 2, while showing low expression of approximately −2 in areas 3, 4, and 5. *Hydra-*associated *Curvibacter* on the other hand showed strong negative expression levels with Z-scores up to −3 in areas 1 and 2, while other areas were highly expressed with Z-scores of 1 to 2 (Figure 5D).

In order to investigate differences within the aforementioned lifestyles of *Curvibacter* sp. AEP1.3 at transcriptomic level, we identified differentially expressed transcripts and sorted their predicted products into functional categories. That way we were able to investigate which categories of predicted *Curvibacter* sp. AEP1.3 proteins changed the most depending on substrate. The largest fraction of differentially expressed genes in *Curvibacter sp.* belonged to proteins associated with the plasma membrane, with a share of 43.88% among a total of 1,169 proteins. The second largest fraction consisted of cytosolic proteins at 26.43%, followed by proteins located in the periplasmic space at 5.22%, ATP-binding cassettes at 4.96%, and extracellular proteins with a share of 1.54%. These results indicated that both extracellular proteins and plasma membrane-associated proteins were most affected by changes in substrate, as they made up more than half of all differentially expressed proteins (Figure 5E).

### Identification of phage-binding protein candidates

With the goal of identifying potential phage-binding protein candidates in mind, we ranked our differentially expressed genes by log_2_ fold changes (log_2_ fc) converted into Z-scores, to select the most differentially expressed genes in *Curvibacter* sp. AEP1.3 on solid medium compared to both other states. The resulting list was led by a PKHD-type hydrolase with a fold change of 3.03, followed by several metabolic proteins that performed glycine cleavage and xylose uptake. Since we were looking for phage-binding candidates, we focused on surface proteins rather than intracellular proteins, whereas the putative TonB-dependent receptor BfrD with a fold change of 2.44 and the Methyl-accepting chemotaxis protein II with a fold change of 1.08 stood out as particularly interesting candidates (Figure 5F).

To complete the picture and find potential inhibitors of phage infection, we investigated which proteins were expressed the least in sessile *Curvibacter* sp. AEP1.3, by sorting them *via* their Z-scores. A tRNA modifying GTPase was expressed the least, with a Z-score of −1.31, followed by

O-acetyltransferases, multiple proteases, and toxin export proteins. Particularly noteworthy was the downregulation of a CRISPR Cas3 subtype with a Z-score of −1.17 (Figure 5G).

## Discussion

### PCA1 infectivity and potential phage-binding candidates

This study started with the intent of eliminating *Curvibacter sp.* AEP1.3 on *Hydra* using bacteriophages and thus investigating the impact of losing a highly abundant beneficial colonizer. We selected the *Curvibacter* phage PCA1 for this purpose because of its narrow host range and lack of ability to infect other bacterial strains associated with *Hydra vulgaris* AEP (Figure 1C). Initial steps proceeded as expected, since we were able to identify the phage, observe plaque formation and propagate PCA1 phages on plate (Figure 1D). PCA1 phage’s inability to infect *Curvibacter* sp. AEP1.3 in liquid culture was unexpected (Figure 1E). While it has been recorded that community composition affected phage susceptibility (Blazanin and Turner, 2021) literature has indicated that solid bacterial aggregates hindered phage propagation due to perturbed diffusion, fibrous barriers, and increased nutrient availability (Lourenço et al., 2018; Sousa and Rocha, 2019) and that contrary to our findings, bacteria were found to be more susceptible to phages in liquid culture (Testa et al., 2019). Since evidence of a phage that only infects bacteria on solid medium and not in liquid culture is rare, we chose to investigate this phenomenon in greater detail.

After we were able to exclude technical artifacts such as temperature and nutrient supply, the addition of glass fiber allowed us to narrow down the cause behind *Curvibacter* sp. AEP1.3’s varying susceptibility to a difference in substrate texture (Figure 3A). This observation led us to hypothesize that *Curvibacter* phage PCA1 used cell surface proteins necessary for adhesion to enter *Curvibacter sp.* AEP1.3. This hypothesis was further supported as plasma membrane proteins made up more than half of all differentially expressed proteins (Figure 5E). Since plasma membrane proteins were recorded to have a half-life ranging from 15 min up to 6 days (Ito and Akiyama, 2005; Chai et al., 2016), our hypothetical membrane-associated phage receptor should still be present after transferring *Curvibacter sp.* AEP1.3 from solid medium to liquid culture. As a consequence, bacteria transferred from solid to liquid medium should still be susceptible to infection. Instead, we observed that *Curvibacter sp.* AEP1.3 were immediately immune to phage infection (Figure 3B). Only when we combined *Curvibacter sp.* AEP1.3 from a plate with supernatant from sessile *Curvibacter*, was phage susceptibility returned. This indicates that at least two separate factors were required to achieve phage infection, such as presence of a membrane protein together with a specific signal. This would exclude porins and other permanently open channels from our list of candidates for phage-binding proteins.

Our reason for not considering signaling molecules on their own or a quorum sensing-based mechanism as cause of phage susceptibility is, that addition of supernatant from plated AEP1.3 should have been sufficient if we only required a signaling molecule to induce phage infection. Another argument is that the hypothetical phage receptor may have been degraded rapidly, but then we would not have been able to see a resurgence in infectivity after adding supernatant to sessile *Curvibacter* sp. AEP1.3 (Figure 3D), unless the hypothetical phage receptor was degraded rapidly and had to be produced again. If supernatant addition was indeed responsible for inducing production of our hypothetical phage receptor, it would explain the curious delay of 12 h, during which we observed no phage infection (Figure 3C). If that were the case, however, addition of supernatant alone would again have been sufficient to induce phage infection. In conclusion, the fact that both supernatant and previously sessile *Curvibacter* sp. AEP1.3 were required to induce phage infection (Figure 3D), indicating that we should look for a system consisting of a plasma membrane protein with a secondary, functionally linked component, when searching for a candidate protein.

Among top differentially expressed proteins in sessile *Curvibacter sp.* we found multiple interesting candidates. We consider putative TonB-dependent receptor BfrD to be the most likely candidate for PCA1 phage binding. This hypothesis is based on differential expression of TonB, as it was upregulated in sessile *Curvibacter sp.* AEP1.3 and downregulated in *Curvibacter* on *Hydra* and liquid medium, respectively. Congruously, TonB receptors are multiple component systems with a plug domain that can change conformation to permit or deny translocation (Moynié et al., 2019), which would align well with our previous conclusion of requiring at least two components out of which one has to be a plasma membrane protein. Last but not least, TonB receptors were found to act as phage receptors for bacteriophage H8 in *Salmonella enterica* (Rabsch et al., 2007) and have thus already been shown to allow bacteriophages entry into bacterial cells. Other potential phage-binding candidates include the methyl-accepting chemotaxis protein II, which was also upregulated in sessile *Curvibacter*, while it was downregulated in other expression states. The protein was found to regulate taxis in *Salmonella typhimurium*, where it transduced extracellular signals to the cell’s interior (Cooper et al., 2021). Due to its location in the plasma membrane, it could also function as a receptor for the PCA1 phage.

Downregulation of a CRISPR Cas3 system subtype I-F stands out as well when looking at differential expression of proteins in sessile *Curvibacter,* as it was furthermore upregulated in liquid culture and on *Hydra.* Since CRISPR systems are commonly known to protect bacteria from viral infection (Makarova and Koonin, 2015; Ishino et al., 2018), expression of this Cas3 subtype in liquid culture and on *Hydra,* together with downregulation in sessile *Curvibacter* could indicate a lack of defense in sessile *Curvibacter* that could result in its susceptibility to phages on solid medium. On the other hand, it seems that this particular CRISPR system is most similar to one found in *P. aeruginosa PA14*, wherein the Cas3 1-F subtype was responsible for suppressing biofilm formation rather than playing a role in antiviral defense (Heussler et al., 2015). Another argument against phage defense by *Curvibacter* sp. AEP1.3 is that PFU counts remain similar after incubation in liquid culture (Figure 3A), when we would at least expect a decrease in PFU, if *Curvibacter* were to actively destroy phages. Thus, we retain that the putative TonB-dependent receptor BfrD is the most likely candidate responsible for deciding phage binding and infection.

### *Curvibacter* sp. AEP1.3’s adaptive lifestyle and its implications

Another important takeaway from our study is the fact that *Curvibacter* phage PCA1 does not simply infect *Curvibacter sp.* AEP1.3 regardless of environment. While this may seem like a trivial statement, its implications for the usage of phages in phage therapy and microbiota research are severe. Treatment could for example require different phages depending on whether a bacterial infection is situated in the human intestine, similar to *Hydra’s* mucus layer (Schröder and Bosch, 2016), or whether bacteria have become sessile on a skin lesion. While we found a phage that only infects in solid medium, it is not farfetched to hypothesize that phages exist which solely infect host-associated or planktonic bacteria. A phage’s suitability for treatment would have to be tested on bacteria in the same expression state as those bacteria in need of treatment. In order to adjust our frame of reference accordingly, we need to come up with a system to define these different states.

One way to distinguish between expression states would be to divide them into two, as we had hypothesized after observing infection in sessile but not in planktonic *Curvibacter* sp. AEP1.3. A similar approach to describe microbe-host interactions has already been taken by Obeng et al. (2021), who proposed to differentiate between free-living and host-associated members of the same species based on differences in fitness. They referred to such microbial behavior as a biphasic life cycle. Evidence of a biphasic life cycle has been found in *B. bacteriovorus, where significant metabolic changes were observed when comparing predating bacteria with* intraperiplasmic bacteria of the same strain (Herencias et al., 2020) and in virulent versus non-virulent *L. pneumophila* (Ge et al., 2022).

We want to build on this concept by adding one more state, resulting in a model that differentiates between free-living (planktonic), host-associated, and sessile bacteria (Figure 5A) based on protein expression patterns. In *Curvibacter sp.* AEP1.3, sessile bacteria were able to latch onto solid substrates to the point that attachment to a substrate (Figures 1D, 3A) was sufficient to warrant 24% difference in comparison to *Curvibacter sp.* in liquid R2A culture, even though other factors like nutrients and temperature were the exact same as on R2A agar plates. *Curvibacter sp.* AEP1.3 on *Hydra* likely showed a larger difference compared to *Curvibacter* in liquid culture and on solid medium, since *Curvibacter* on *Hydra* were exposed to changes both in substrate texture and nutrient environment (Figure 5B). While we show three distinct cases of *Curvibacter* adapting its gene expression to environmental conditions, including surface structure and nutrient conditions, we expect that *Curvibacter’s* adaptive lifestyle is not limited to these three and may further respond according to changing environments. Lastly, we can conclude that while bacteria have been noted to form biofilms on their host (Pires et al., 2021) *Curvibacter sp.* AEP1.3 are not even sessile on *Hydra vulgaris* AEP. The first indicator was that bacterial cells floated freely in the mucus surrounding Hydra (Figure 4B) rather than forming a solid film, which was further supported by *Curvibacter* phage PCA1 not infecting *Curvibacter sp.* AEP1.3 on *Hydra* in the same way it did not infect planktonic *Curvibacter.* We were able to obtain further evidence of this from distinct expression patterns of *Curvibacter* on *Hydra* in principal component analysis (Figure 5B), as it showed a unique expression pattern compared to sessile *Curvibacter sp.* AEP1.3. For future host-microbiota research, we need to keep in mind that these changes in expression pattern cannot be discerned by metagenome sequencing alone. In order to grasp the full breadth of interactions between a host and its microbiota, we may need to add RNA sequencing to our array of tools more frequently.

## Data availability statement

The datasets presented in this study can be found in online repositories. The phage genome presented in this study can be found at GenBank, accession number: BankIt2629455 Seq1 OP588919. The RNA sequences can be found at the sequencing read archive (https://www.ncbi.nlm.nih.gov/sra/PRJNA887579), bioproject number: PRJNA887579.

## Author contributions

LU and TL performed host range assays and growth experiments. LU performed experiments on Hydra, extracted and analyzed RNA sequences, wrote this manuscript, and visualized data. TL isolated the PCA1 phage, performed TEM, and supervised this project. CG also did RNA extraction, genetically modified *Curvibacter,* and wrote the respective methods sections. LXS analyzed the phage genome, annotated it, and wrote respective sections, including Figure 2 and Supplementary Figure S1. All authors contributed to the article and approved the submitted version.

## Funding

This research was funded by the Deutsche Forschungsgemeinschaft (DFG, German Research Foundation) Project-ID 261376515 – CRC 1182 “Origin and function of metaorganisms,” Project C4.2: “Phage regulated, rapid acclimatization of *Hydra*.”

## Conflict of interest

The authors declare that the research was conducted in the absence of any commercial or financial relationships that could be construed as a potential conflict of interest.

## Publisher’s note

All claims expressed in this article are solely those of the authors and do not necessarily represent those of their affiliated organizations, or those of the publisher, the editors and the reviewers. Any product that may be evaluated in this article, or claim that may be made by its manufacturer, is not guaranteed or endorsed by the publisher.

## Footnotes

^1^ https://vogdb.org/

## Supplementary material

SUPPLEMENTARY FIGURE S1 Taxonomic Tree comparing the PCA1 phage to 200 related phages based on proteomic similarity. The left box indicates the viral family with Siphoviridae in orange, Myoviridae in green and Podoviridae in blue, while the right box indicates the class of bacterial hosts with Gammaproteobacteria in green, Actinobacteria in cyan, Betaproteobacteria in brown, Firmicutes in purple, Cyanobacteria in red and others in dark gray.

SUPPLEMENTARY FIGURE S2 **(A)** Overlay agar with *Curvibacter* phage PCA1 spotted on top and mixed into *Curvibacter sp.* AEP1.3. **(B)** Growth curves of *Curvibacter sp.* AEP1.3 with and without *Curvibacter* phage PCA1 measured at OD600. Magnesium sulfate, magnesium chloride, and calcium chloride were added respectively, **(C)** Growth curves of *Curvibacter sp.* AEP1.3 with and without *Curvibacter* phage PCA1 measured at OD600. Ethyl acetate and 3-oxo-homoserine lactones (3OHC12 and 3OC12) were added, respectively. **(D)** Top 10 overexpressed and underexpressed proteins in *Curvibacter sp.* AEP1.3 on *Hydra vulgaris* AEP, sorted by Z-scores of log_2_ fold changes. Top overexpressed and top underexpressed proteins in *Curvibacter sp.* AEP1.3 on *Hydra vulgaris* AEP after RNA Seq. according to Z-scores. **(E)** Growth curves showing *Curvibacter sp.* AEP1.3 transferred from solid medium to liquid culture, measured at OD_600_. Supernatant from plated *Curvibacter sp.* AEP1.3 was divided into a small fraction and large fraction before being added to treatments in addition to PCA1 phage.
