# Supplementary figures and images for: Adaptive lifestyle of bacteria determines phage-bacteria interaction
Source: Front Microbiol. 2022 Dec 6;13:1056388. doi: 10.3389/fmicb.2022.1056388 (PMC9763317; doi:10.3389/fmicb.2022.1056388)

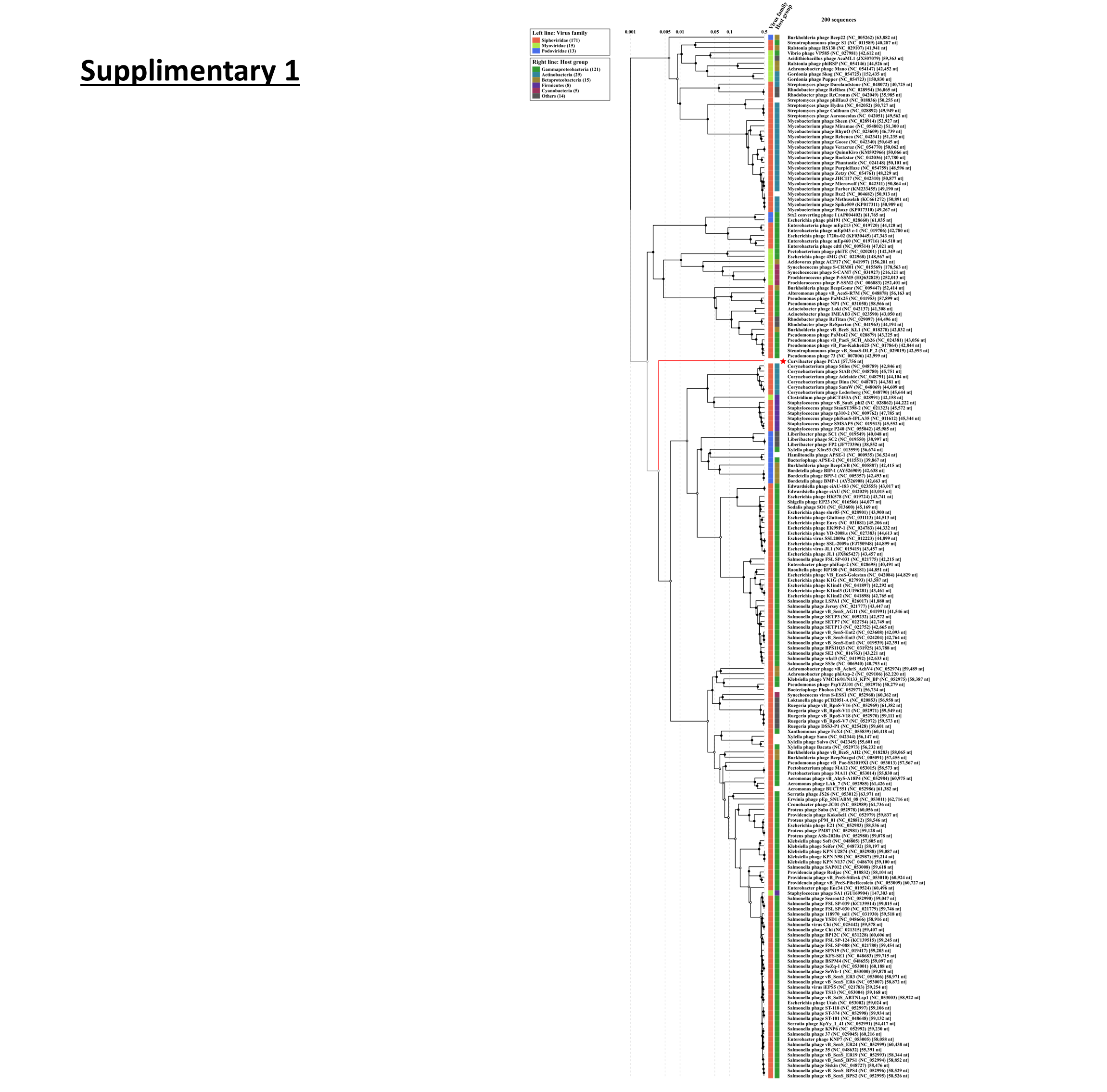

Supplement: SUPPLEMENTARY FIGURE S1 — Taxonomic Tree comparing the PCA1 phage to 200 related phages based on proteomic similarity. The left box indicates the viral family with Siphoviridae in orange, Myoviridae in green and Podoviridae in blue, while the right box indicates the class of bacterial hosts with Gammaproteobacteria in green, Actinobacteria in cyan, Betaproteobacteria in brown, Firmicutes in purple, Cyanobacteria in red and others in dark gray. [file Image_1.TIF]

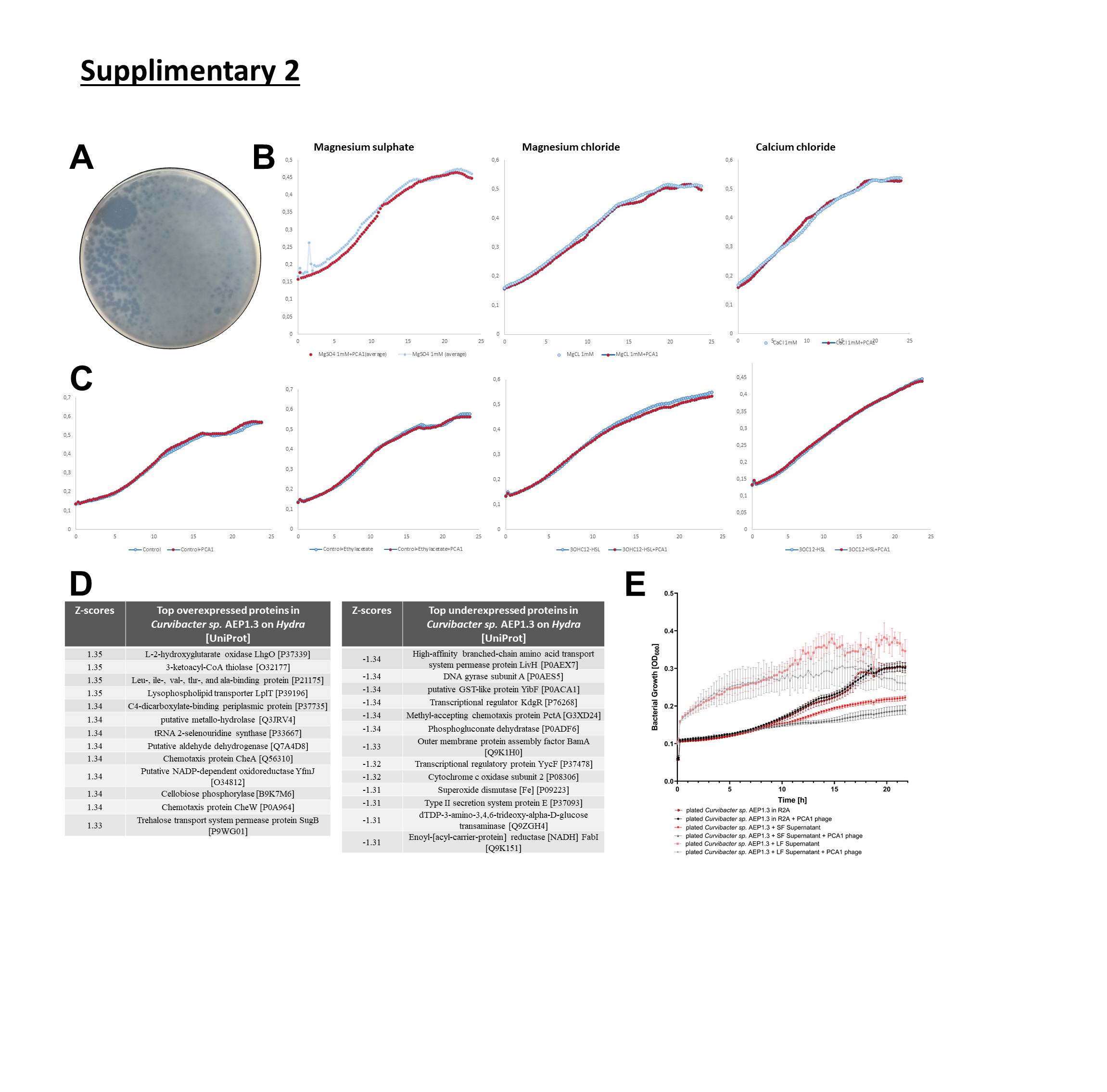

Supplement: SUPPLEMENTARY FIGURE S2 — (A) Overlay agar with Curvibacter phage PCA1 spotted on top and mixed into Curvibacter sp. AEP1.3. (B) Growth curves of Curvibacter sp. AEP1.3 with and without Curvibacter phage PCA1 measured at OD600. Magnesium sulfate, magnesium chloride, and calcium chloride were added respectively, (C) Growth curves of Curvibacter sp. AEP1.3 with and without Curvibacter phage PCA1 measured at OD600. Ethyl acetate and 3-oxo-homoserine lactones (3OHC12 and 3OC12) were added, respectively. (D) Top 10 overexpressed and underexpressed proteins in Curvibacter sp. AEP1.3 on Hydra vulgaris AEP, sorted by Z-scores of log2 fold changes. Top overexpressed and top underexpressed proteins in Curvibacter sp. AEP1.3 on Hydra vulgaris AEP after RNA Seq. according to Z-scores. (E) Growth curves showing Curvibacter sp. AEP1.3 transferred from solid medium to liquid culture, measured at OD600. Supernatant from plated Curvibacter sp. AEP1.3 was divided into a small fraction and large fraction before being added to treatments in addition to PCA1 phage. [file Image_2.TIF]
